# Expression of Tissue Factor and Platelet/Leukocyte Markers on Extracellular Vesicles Reflect Platelet–Leukocyte Interaction in Severe COVID-19

**DOI:** 10.3390/ijms242316886

**Published:** 2023-11-28

**Authors:** Tanja Eichhorn, René Weiss, Silke Huber, Marie Ebeyer-Masotta, Marwa Mostageer, Robert Emprechtinger, Ludwig Knabl, Ludwig Knabl, Reinhard Würzner, Viktoria Weber

**Affiliations:** 1 Center for Biomedical Technology, Department for Biomedical Research, University for Continuing Education Krems, 3500 Krems, Austria; rene.weiss@donau-uni.ac.at (R.W.); marie.ebeyer-masotta@donau-uni.ac.at (M.E.-M.); marwa.mostageer@donau-uni.ac.at (M.M.); viktoria.weber@donau-uni.ac.at (V.W.); 2 Institute of Hygiene and Medical Microbiology, Medical University of Innsbruck, 6020 Innsbruck, Austria; silke.huber@i-med.ac.at (S.H.); reinhard.wuerzner@i-med.ac.at (R.W.); 3 Faculty of Health and Medicine, University for Continuing Education Krems, 3500 Krems, Austria; emprechtinger@stateofhealth.at; 4 Department of Internal Medicine, Hospital St. Vinzenz, 6511 Zams, Austria; aon.964003992.knabl@aon.at; 5 TyrolPath, 6511 Zams, Austria; ludwig.knabl@tyrolpath.at

**Keywords:** platelet–leukocyte aggregates, extracellular vesicles, tissue factor, COVID-19, immunothrombosis

## Abstract

Severe COVID-19 is frequently associated with thromboembolic complications. Increased platelet activation and platelet–leukocyte aggregate formation can amplify thrombotic responses by inducing tissue factor (TF) expression on leukocytes. Here, we characterized TF-positive extracellular vesicles (EVs) and their cellular origin in 12 patients suffering from severe COVID-19 (time course, 134 samples overall) and 25 healthy controls. EVs exposing phosphatidylserine (PS) were characterized by flow cytometry. Their cellular origin was determined by staining with anti-CD41, anti-CD45, anti-CD235a, and anti-CD105 as platelet, leukocyte, red blood cell, and endothelial markers. We further investigated the association of EVs with TF, platelet factor 4 (PF4), C-reactive protein (CRP), and high mobility group box-1 protein (HMGB-1). COVID-19 patients showed higher levels of PS-exposing EVs compared to controls. The majority of these EVs originated from platelets. A higher amount of EVs in patient samples was associated with CRP, HMGB-1, PF4, and TF as compared to EVs from healthy donors. In COVID-19 samples, 16.5% of all CD41^+^ EVs displayed the leukocyte marker CD45, and 55.5% of all EV aggregates (CD41^+^CD45^+^) co-expressed TF, which reflects the interaction of platelets and leukocytes in COVID-19 on an EV level.

## 1. Introduction

Thrombotic events and endothelial dysfunction are key pathomechanisms of coronavirus disease-19 (COVID-19) caused by the severe acute respiratory syndrome coronavirus 2 (SARS-CoV-2) [1,2]. In severe COVID-19, deregulated complement activation enhances the recruitment of neutrophils to the infected lungs and promotes tissue factor (TF) expression on neutrophils, monocytes, and endothelial cells, resulting in microvascular thrombosis and endothelial dysfunction [1,3,4]. These events are best described as immunothrombosis, a simultaneous overactivation of the innate immune response and the coagulation system [5]. Activated platelets are main effectors of immunothrombosis and interact with leukocytes to promote pro-inflammatory and pro-coagulant responses [4]. Platelets from COVID-19 patients show a hyper-reactive phenotype characterized by the release of platelet factor 4 (PF4), platelet-derived growth factor (PDGF), and thromboxane A2, as well as increased P-selectin expression, which mediates platelet–leukocyte aggregate formation via P-selectin glycoprotein ligand (PSGL)-1 [6,7,8,9,10]. The enhanced aggregate formation of platelets with monocytes or neutrophils in COVID-19 has been correlated with a poor prognosis [7,9,11].

The platelet–leukocyte crosstalk can boost the dysregulated cytokine and chemokine response seen in COVID-19 and triggers TF expression on monocytes, neutrophils, and endothelial cells [6]. Moreover, aggregate formation of platelets with monocytes drives TF expression on monocytes via P-selectin and integrin αIIb/β3-dependent signaling and is amplified by TNF-α and IL-1β [12]. Likewise, platelets can induce TF expression on neutrophils and promote the release of neutrophil extracellular traps (NETs), representing a scaffold of expelled nuclear DNA and histones that captures and kills pathogens via antimicrobial proteins and proteolytic enzymes [13]. NETs support the pro-thrombotic phenotype via their ability to initiate coagulation, either by presenting TF [14] or by triggering the contact-dependent pathway via factor XII [15]. NETs promote platelet binding and the formation of platelet–neutrophil aggregates, which fuel further NET release and thus exacerbate endothelial damage and thrombotic complications [6,16,17]. Upon activation, platelets as well as neutrophils and monocytes release extracellular vesicles (EVs) into the circulation, which are important mediators of thrombosis due to their exposure of phosphatidylserine (PS) [18,19,20] and TF [21,22,23,24,25]. Furthermore, EVs exert immunomodulatory functions by transferring regulatory and pro-inflammatory molecules, such as nucleic acids, C-reactive protein (CRP), or high mobility group box-1 protein (HMGB-1) [26,27,28]. 

Here, we characterized the cellular origin of PS-exposing EVs in plasma from COVID-19 patients as well as their association with inflammation- and coagulation-related parameters, including CRP, HMGB-1, PF4, and TF. We identified TF-bearing EVs co-expressing platelet (CD41) and leukocyte (CD45) markers, reflecting the recently described platelet–leukocyte aggregate formation in severe COVID-19 on the EV level.

## 2. Results

### 2.1. Patient Characteristics

**Figure 1:**
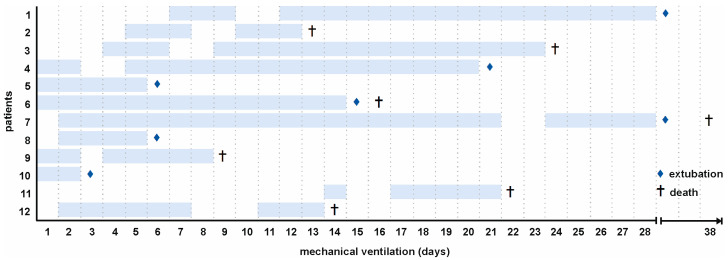
Timeline of sampling. Samples from 12 COVID-19 patients were analyzed in this study (134 samples in total). Sampling was initiated on the day of intubation. Blue frames indicate sampling, (♦) marks extubation, and (†) death of patients.

In total, 12 critically ill COVID-19 patients receiving mechanical ventilation were included in this study. Twenty-five healthy individuals served as controls. Baseline characteristics and comorbidities are summarized in Table 1. The medication regimens for individual patients are detailed in Supplementary Table S1. The mean age of COVID-19 patients was 79.5 [77.0–82.9] years, and the majority of patients were males (83%). Secondary bacterial (*E. coli*) and/or fungal (*Aspergillus fumigatus*) infections were confirmed in 58% of all patients. The overall mortality was 58%. The time points of sample collection for individual patients are shown in Figure 1.

### 2.2. Cellular Origin of Extracellular Vesicles

**Figure 2:**
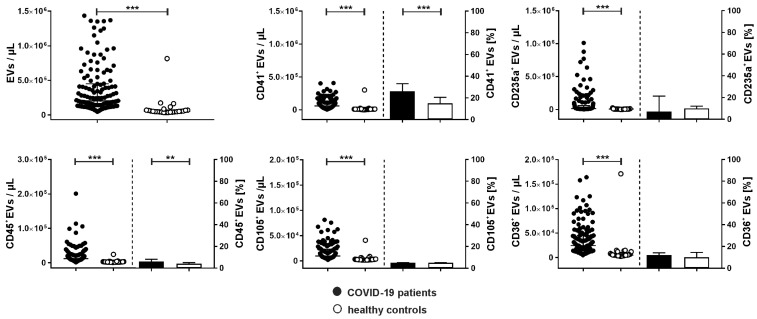
Cellular origin of extracellular vesicles in COVID-19 patients. Extracellular vesicles in COVID-19 patients (n = 134; black) and healthy controls (n = 25; white) were characterized by flow cytometry and stained by a combination of anti-CD41 as platelet marker, anti-CD235a as red blood cell marker, anti-CD45 as leukocyte marker, anti-CD105 as endothelial marker, and anti-CD36 to detect platelet glycoprotein IV as described in Section 4. Annexin V (Anx5) was used as marker for EVs exposing phosphatidylserine. Data are given as median [IQR; interquartile range] and were compared using the Mann–Whitney test (** *p* < 0.01, *** *p* < 0.001).

COVID-19 patients showed significantly increased levels of PS-exposing circulating EVs compared to healthy donors (204,000 [122,375–450,000] vs. 53,500 [43,750–70,500] EVs/µL, Figure 2). These EVs were derived from platelets (CD41^+^; 59,465 [25,980–112,155] vs. 9000 [5850–10,913] EVs/µL), red blood cells (CD235a^+^; 12,385 [7266–57,816] vs. 5400 [4193–7985] EVs/µL), leukocytes (CD45^+^; 11,958 [7359–22,659] vs. 2450 [1830–3378] EVs/µL), and endothelial cells (CD105^+^; 9598 [5843–22,898] vs. 2400 [1788–3335] EVs/µL; see also Supplementary Figure S1 for the time course of EV concentrations). Regarding the relative abundance of PS-exposing EVs in COVID-19 patients, 26.0 [16.0–33.0] % originated from platelets, 7.0 [4.0–21.3] % from red blood cells, 6.0 [4.0–8.0] % from leukocytes, and 5.0 [4.0–5.0] % from endothelial cells. The remaining EVs were of undefined origin as they neither carried CD41, CD45, CD235a, nor CD105 on their surface. CD36, which has been linked to platelet hyperactivation [29,30], was expressed in 12 [9.0–14.0] % of all EVs. 

### 2.3. Association of Extracellular Vesicles with CRP, PF4, and HMGB-1

**Figure 3:**
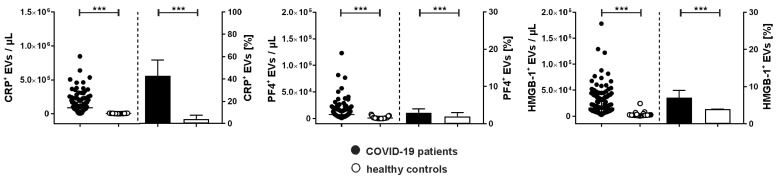
Association of extracellular vesicles with CRP, PF4, and HMGB-1 in COVID-19 patients. Characterization of EVs carrying C-reactive protein (CRP^+^), platelet factor 4 (PF4^+^), and high mobility group box-1 protein (HMGB-1^+^) in plasma samples from COVID-19 patients (n = 134; black) in comparison to healthy controls (n = 25; white) using flow cytometry. Data are given as median [IQR] and were compared using the Mann–Whitney test (*** *p* < 0.001).

Next to the characterization of their cellular origin, we assessed the association of EVs with CRP, PF4, and HMGB-1, which are mediators of immunothrombosis (Figure 3). COVID-19 patients showed significantly increased levels of CRP^+^ EVs, which represented 43.0 [19.0–57.0] % of the total count of PS-exposing EVs, vs. 4.0 [3.0–7.5] % in healthy controls.

Albeit only a small percentage of EVs was associated with PF4 (COVID-19, 3.0 [3.0–4.0] %; healthy controls, 2.0 [0.5–3.0] %), PF4^+^ EVs were significantly elevated in COVID-19 patients compared to healthy donors (7525 [3878–16,385] vs. 1050 [170–2280] EVs/µL). 

HMGB-1 was also detected on EVs. Compared to healthy controls, COVID-19 patients had significantly higher concentrations of HMGB-1^+^ EVs (15,443 [8548–36,240] vs. 1980 [1500–2688] EVs/µL).

### 2.4. Tissue Factor Expression on Platelet- and Leukocyte-Derived Extracellular Vesicles

**Figure 4:**
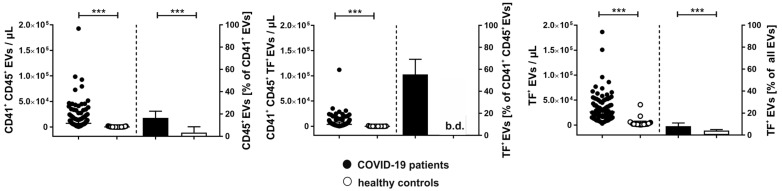
Aggregate formation between platelet-derived and leukocyte-derived EVs and TF expression on EVs in COVID-19 patients. Extracellular vesicles in COVID-19 patients (n = 134; black) and healthy controls (n = 25; white) were characterized by flow cytometry. Aggregates of platelet- and leukocyte-derived EVs were identified as CD41^+^CD45^+^ events in the EV gate as described in Section 4 (**left**: EV counts/µL and the percentage of platelet EVs carrying CD45 as leukocyte marker are shown). A total of 55.5% of CD41^+^CD45^+^ EVs in COVID-19 patients expressed TF (**middle**). Overall, TF expression on EVs was significantly enhanced in COVID-19 patients (**right**). Data are given as median [IQR] and were compared using the Mann–Whitney test (*** *p* < 0.001); b.d., below detection.

In COVID-19 patients, 16.5 [9.0–22.5] % of all platelet EVs were CD41^+^ and CD45^+^, providing evidence for aggregate formation between platelet- and leukocyte-derived EVs, whereas only 3.4 [1.6–8.5] % of CD41^+^CD45^+^ EVs were detected in healthy donors (Figure 4, left panel). Remarkably, 55.5 [42.9–69.2] % of all EV aggregates (CD41^+^CD45^+^) in COVID-19 patients co-expressed TF, while the percentage of TF^+^ EV aggregates remained below the detection limit in healthy donors (Figure 4, middle panel). For individual patients, the percentages of CD41^+^CD45^+^TF^+^ EVs ranged from 25.0% to 76.5% (Supplementary Figure S2). Overall, EVs from COVID-19 patients expressed TF to a significantly higher extent than EVs from healthy donors (18,925 [10,053–33,028] vs. 1880 [1335–3945] EVs/µL, respectively). In the COVID-19 group, 8.0 [6.0–11.0] % of all EVs (which were defined as Anx5^+^ events in the EV gate as described in Section 4.2) were associated with TF as compared to 4.0 [2.0–5.0] % in healthy donors (Figure 4, right panel). TF^+^ EVs correlated with soluble PF4 (r = 0.5282, *p* < 0.001). No correlation of TF^+^ EVs with D-dimer or nucleosomes was detected. The baseline levels of TF^+^ EVs did not differ between survivors and non-survivors. However, the small number of patients did not allow for a correlation to clinical endpoints. 

### 2.5. Time Course of Coagulation- and Inflammation-Related Mediators 

Statistical modeling of the time effect on coagulation- and inflammation-related mediators revealed a decrease over time in leukocytes, neutrophils, platelets, PF4, EV counts, CRP^+^ EVs, TF^+^ EVs, and IP-10 in COVID-19 patients with a probability of >95% (Table 2; see also Supplementary Figure S3 for the time course of inflammatory mediators). IL-8, IL-1β, granulocyte colony-stimulating factor (G-CSF), nucleosomes, TNF-α, D-dimer, IL-10, and HMGB-1 levels increased over time with a probability in the range of 100.0–92.7%.

## 3. Discussion

Immunothrombosis, the joint overactivation of the innate immune response and coagulation, is a central pathomechanism in sepsis and severe COVID-19. Complement activation and cytokine release, platelet hyperactivity, as well as coagulopathy play critical roles in this complex scenario [31,32]. In particular, the crosstalk of platelets and leukocytes has been shown to amplify inflammatory effector functions, as discussed in more detail below [9,17].

Increased levels of circulating EVs released from activated blood cells are well documented in sepsis and severe COVID-19 [33]. EVs released from the plasma membrane expose PS and support coagulation by catalyzing the formation of the tenase and pro-thrombinase complexes of the coagulation cascade [19]. Therefore, we focused on the characterization of this EV population in our study. Our data confirm the presence of increased levels of circulating PS-exposing EVs in patients with severe COVID-19. In line with previous reports [10,24,34,35], the majority of these EVs originated from platelets. 

While flow cytometry is versatile and well established to characterize EVs in plasma samples, the limitations of this approach have to be taken into account. There is evidence that Anx5, which we used to detect PS-exposing EVs in this study, also labels apolipoprotein B-containing lipoproteins, such as low-density lipoprotein [36], challenging the use of Anx5 to uniquely identify EVs in lipoprotein-containing samples. This is why we combined the PS-based approach for EV detection with additional membrane-bound markers (CD41, CD45, CD235a, and CD105), which are not exposed on lipoproteins. Moreover, the fact that we compared TF expression on EVs in patient samples to samples from healthy donors greatly limits a potential bias in our results, since lipoproteins would have been present in the control samples as well. 

We found that EVs from COVID-19 patients carry CRP, PF4, and HMGB-1 on their surface, which may further fuel immunothrombosis. Elevated levels of CRP^+^ EVs have been described in sepsis but also in myocardial infarction, and several studies have highlighted the pro-inflammatory characteristics of these CRP-bearing EVs [26,37,38]. Sustained platelet activation in COVID-19 boosts the release of PF4 and HMGB-1, inducing neutrophil activation and the release of NETs, which strongly promote coagulation [4,10,17,39]. Platelet-derived HMGB-1^+^ EVs have been reported as markers of platelet activation and are associated with a poor prognosis in COVID-19 patients [27]. Furthermore, we found increased levels of CD36^+^ EVs in COVID-19 patients. Platelet glycoprotein CD36 acts as a receptor for membrane-derived EVs, as it binds to PS on their surface [30], resulting in platelet activation, aggregation, and thrombus formation [29]. Accordingly, increased CD36 expression indicates a higher risk of venous and arterial thromboembolism [40].

Next to characterizing PS-exposing EVs, which propagate coagulation, as discussed above, we focused on the expression of TF on EVs, which is the main initiator of coagulation. Monocytes represent the predominant source of blood-borne TF, which can be passed on to EVs originating from these cells [41,42]. While we were not able to obtain samples for all time points from each COVID-19 patient due to extubation or death, our data show that each patient had increased levels of TF^+^ EVs at all available time points in comparison to healthy controls. While several previous studies have also reported increased levels of TF-expressing EVs in COVID-19 patients, which correlated with disease severity [21,24,42], others failed to detect differences in TF^+^ EVs between COVID-19 patients and healthy controls [35]. These conflicting results may, at least in part, be attributed to the different antibody clones used for the detection of TF or to the different fluorochrome-to-protein ratios of antibody–fluorochrome conjugates used for the flow cytometric detection of TF^+^ EVs. 

Enhanced platelet–leukocyte aggregate formation is known to occur in various pathological conditions, including sepsis and COVID-19, where increased platelet–monocyte interaction has been linked to disease severity [39]. Our data suggest that the enhanced interaction of platelets and leukocytes in COVID-19 is also reflected at the EV level. About 17% of all platelet-derived EVs displayed the leukocyte marker CD45, suggesting aggregate formation between platelet-derived and leukocyte-derived EVs. Notably, 55.5% of all CD41^+^CD45^+^ EVs expressed TF, likely of monocyte or neutrophil origin. As a limitation, our flow cytometric protocol based on the pan leukocyte marker CD45 did not allow for the discrimination between monocyte-derived and neutrophil-derived EVs, to further characterize the source of blood-borne TF. 

It is well established that activated platelets express P-selectin and interact with monocytes through PSGL-1 [43], a mechanism that may also mediate aggregate formation between platelet- and leukocyte-derived EVs. Platelets induce TF expression on monocytes through P-selectin and integrin αIIb/β3 signaling [39] and stimulate the release of TF-conveying NETs by neutrophils [44]. In turn, increased TF expression has been associated with an upregulation of CD16 on monocytes, inducing a shift from CD16^–^ classical monocytes, which are mainly phagocytic, towards inflammatory CD16^+^ intermediate and non-classical monocytes [12]. 

Platelet-monocyte aggregates also promote the secretion of inflammatory mediators. Incubation of platelets from COVID-19 patients with monocytes from healthy donors triggers the release of IL-1β, IL-6, IL-8, MIP-1α, MCP-1, TNF-α, PF4, and PDGF [12,39], and elevated levels of these factors were observed in clinical samples from COVID-19 patients [45]. Likewise, our study cohort presented increased levels of inflammatory mediators that dynamically changed during the course of mechanical ventilation, with an increase in IL-8, IL-1β, G-CSF, nucleosomes, TNF-α, D-dimer, IL-10, and HMGB-1 over time. A similar time-dependent increase has been reported for IL-6, IL-8, IL-1β, and TNF-α in COVID-19 [46]. We further observed decreasing platelet, leukocyte, and neutrophil counts in mechanically ventilated COVID-19 patients over time. 

To conclude, we report here for the first time that TF-expressing platelet- and leukocyte-derived EV aggregates are present in severely ill COVID-19 patients, and we propose that these aggregates may act as amplifiers of immunothrombosis.

## 4. Materials and Methods

### 4.1. Patients and Sample Collection

Twelve patients with PCR-confirmed or suspected SARS-CoV-2 infection requiring mechanical ventilation were included in this study at the Department of Internal Medicine, Hospital St. Vinzenz, Zams, Austria, between November 2020 and January 2021 [32]. Sample collection was approved by the ethics committee of the Medical University of Innsbruck (1144/2020). The study was conducted in accordance with the Declaration of Helsinki. Whole blood samples anticoagulated with EDTA (S-Monovette^®^ K3 EDTA, Sarstedt, Nümbrecht, Germany) were obtained during routine blood collection every 24 h. Overall, 134 samples were collected. The control group (n = 25) consisted of healthy individuals from whom EDTA-anticoagulated blood was obtained after written consent. Platelet-poor plasma was obtained by centrifugation of whole blood at 2000× *g* for 15 min at 22 °C and stored at −80 °C until further analysis. Routine laboratory measurements (C-reactive protein, CRP; procalcitonin, PCT; and D-dimer) and blood cell counts were obtained as part of standard medical care. Non-survival of patients was defined as death during mechanical ventilation or within 14 days following extubation. 

### 4.2. Flow Cytometric Characterization of Phosphatidylserine-Exposing Extracellular Vesicles

Due to their pro-coagulant properties, we focused on PS-exposing EVs, which represent a subpopulation of larger EVs derived from the cell membrane (“microvesicles”) and can be detected using Annexin V (Anx5). To avoid interference of lipoproteins, which can also expose PS, we combined Anx5 staining with additional membrane-bound markers (CD41, CD45, CD235a, CD105, and CD36) that are absent on PS-exposing lipoproteins. EVs were characterized by flow cytometry using a CytoFLEX LX device (Beckman Coulter, Brea, CA, USA) equipped with 405 nm, 488 nm, 561 nm, and 631 nm lasers. Calibration of the flow cytometer was performed with fluorescent silica beads (1 μm, 0.5 μm, and 0.1 μm; excitation/emission 485/510 nm; Kisker Biotech, Steinfurt, Germany). The triggering signal for EVs was set to the violet side scatter (405 nm), and the EV gate was set below the 1 µm bead cloud as previously described [47,48]. 

For staining, plasma samples were diluted 1:100 in 0.1 µm sterile-filtered Anx5 binding buffer (BD Biosciences, San Jose, CA, USA). The cellular origin of EVs was assessed by staining of the diluted samples (100 μL each; 30 min; room temperature; in the dark) with a combination of 2 µL PC7-conjugated anti-CD41 (Beckman Coulter) as platelet marker, 2 µL FITC-conjugated anti-CD235a (eBioscience, San Diego, CA, USA) as red blood cell marker, 5 µL PE-conjugated anti-CD105 (Becton Dickinson, Franklin Lakes, NJ, USA) as endothelial marker, 2 µL PB-conjugated anti-CD45 (Beckman Coulter) as leukocyte marker, and 2.5 µL AF700-conjugated anti-hCD36 (BioLegend, San Diego, CA, USA) to detect platelet glycoprotein IV. The association of EVs with coagulation- and inflammation-related mediators was assessed by staining aliquots of the diluted samples (100 µL each; 30 min; room temperature; in the dark) with 1 µL FITC-conjugated anti-CRP (Abcam, Cambridge, UK) to detect CRP, 5 µL AF700-conjugated anti-hHMGB-1 (R&D Systems, Minneapolis, MN, USA) to detect HMGB-1, and 2.5 µL PE-conjugated anti-hCXCL4 (R&D Systems) to detect PF4. Calibration and all controls for the flow cytometric characterization of EVs are shown in Supplementary Figure S4.

The co-expression of TF on EVs of platelet and leukocyte origin was assessed by staining with 2 µL FITC-conjugated anti-hTF (Biomedica Diagnostics, Stamford, CT, USA), 2 µL PC7-conjugated anti-CD41, and 2 µL PB-conjugated anti-CD45 for 30 min at 4 °C in the dark. APC-conjugated Anx5 (2 µL, BD Biosciences) was used as marker for EVs exposing PS. The gating strategy to define TF^+^ EVs is shown in Supplementary Figure S5. Prior to use, all fluorochrome conjugates were centrifuged at 18,600× *g* for 10 min at 4 °C to remove eventual precipitates. All fluorochrome conjugates and the respective antibody clones are listed in Table 3.

Prior to analysis, stained samples were diluted 1:5 in 0.1 µm sterile-filtered Anx5 binding buffer. Acquisition was performed for 2 min at a flow rate of 10 µL/min, and Anx5 positive events in the EV gate were quantified. Data were analyzed using the Kaluza Software 2.1 (Beckman Coulter). Further details on the flow cytometric characterization of EVs are reported according to the MIFlowCyt-EV framework in Supplementary Table S2.

### 4.3. Quantification of Cytokines, Chemokines, and Growth Factors

A total of 27 cytokines, chemokines, and growth factors were analyzed using a magnetic bead array assay (Bio-Plex Pro^TM^ human cytokine 27-plex; Bio-Rad, Vienna, Austria), including: interleukin (IL)-1β; IL-1 receptor antagonist (IL-1ra); IL-2; IL-4; IL-5; IL-6; IL-7; IL-8; IL-9; IL-10; IL-12p70; IL-13; IL-15; IL-17A; interferon-gamma (IFN-γ); tumor necrosis factor-alpha (TNF-α); monocyte chemotactic protein-1 (MCP-1); macrophage inflammatory protein-1 alpha and beta (MIP-1α, MIP-1β); regulated on activation, normal T-cell expressed and secreted (RANTES); eosinophil chemotactic protein (eotaxin); interferon-inducible protein 10 (IP-10); granulocyte colony-stimulating factor (G-CSF); granulocyte-macrophage colony-stimulating factor (GM-CSF); basic fibroblast growth factor (bFGF); platelet derived growth factor (PDGF); and vascular endothelial growth factor (VEGF). Plasma samples were diluted 1:4 with sample diluent and analyzed according to the manufacturer’s instructions.

### 4.4. Quantification of PF4, HMGB-1, and Nucleosomes

Plasma concentrations of PF4 and HMGB-1 were quantified by ELISA (R&D Systems and IBL International, Hamburg, Germany). The cell death detection ELISA (Roche, Mannheim, Germany) was used to determine nucleosome levels in plasma samples.

### 4.5. Statistical Analysis

Statistical analysis was carried out using GraphPad Prism version 9.5.1 (La Jolla, CA, USA). Data are represented as median [IQR; interquartile range]. Groups were compared using the Mann–Whitney test. A value of *p* < 0.05 was considered statistically significant.

To assess the effect of time on different parameters, we used the Bayesian methods due to advantages over the frequentist framework [49,50]. Hierarchical models were created, taking the repeated measures for the individual patients into account. To model the effect of time, we used the individual parameters as outcome variables, as well as time and patient as predictors. To estimate the probability of time being positively or negatively associated with the parameters (indicating an increase or decrease over time, respectively), we used the posterior percentages with positive vs. negative slope values for the predictor time. We used the default priors for this analysis. To estimate whether the change in the parameters was associated with survival, we employed the change in the measure from the first observation as the predictor variable. A logistic model with survival as the outcome variable was created. Again, the patient was used as the grouping variable. Priors for the slope were set to normal, with a mean = 0 and a standard deviation = 3. We used the logit of the slope estimate (transformed to percentages) for the effect of changes in the parameters on survival. The analysis was carried out using R (version 4.2.2). The tidyverse [51] package was used for data wrangling and brms [52] to create the statistical models.

## Figures and Tables

**Table 1:** Patient characteristics at baseline.

	COVID-19 (n = 12)	Survivors (n = 5)	Non-Survivors (n = 7)	Controls (n = 25)
Age, years	79.5 [77.0–82.9]	78.1 [70.6–83.9]	80.6 [77.8–81.5]	29.1 [27.4–42.9]
Female	2 (17%)	1 (20%)	1 (14%)	15 (60%)
Male	10 (83%)	4 (80%)	6 (86%)	10 (40%)
Mortality	7 (58%)			
Secondary infections	7 (58%)			
***Comorbidities, n (%)***				
Cardiovascular	10 (83)	4 (80)	6 (86)	n.a.
Renal/genitourinary	1 (8)	0 (0)	1 (14)	n.a.
Endocrine	2 (17)	1 (20)	1 (14)	n.a.
Metabolic	6 (50)	2 (40)	4 (57)	n.a.
Musculoskeletal	1 (8)	1 (20)	0 (0)	n.a.
Psychiatric/mental	2 (17)	2 (40)	0 (0)	n.a.
Autoimmune/immunological	1 (8)	0 (0)	1 (14)	n.a.
Cancer	1 (8)	0 (0)	1 (14)	n.a.

Data are represented as median [interquartile range] and counts (percentage); n.a., not applicable.

**Table 2:** Effect of time on inflammation- and coagulation-related parameters. Probability (P) increase and decrease indicate the probability that a certain parameter will increase or decrease, respectively, depending on time, according to the statistical model. The Estimate refers to the effect size, as an increase of time by one will change the parameter by the Estimate value. The lower and upper 95% credibility intervals (CI) are denoted as CI_lower_ and CI_upper_. As a measure for the model fit, Rhat as well as the Bulk and Tail effective sample size (ESS) are given, confirming acceptable model fit.

Parameter	P_increase_	P_decrease_	Estimate	Estimate Error	CI_lower_	CI_upper_	Rhat	Bulk ESS	Tail ESS
Leukocytes (cells/µL)	0.08	99.92	−153.67	48.1	−246.91	−58.22	1	2344.63	2722.01
Neutrophils (cells/µL)	0	100	−213.65	47.33	−308.98	−118.91	1.01	3150.99	2606.26
Platelets (cells/µL)	0.9	99.1	−7024.9	2941.64	−12,645.01	−1393.28	1	6704.61	2862.77
PF4 (ng/mL)	0	100	−53.12	9.13	−70.53	−35.17	1	3299.33	3140.46
D-dimer (ng/L)	95.97	4.03	0.1	0.06	−0.02	0.2	1	2889.52	2843.01
Nucleosomes (AU)	99.22	0.78	0.01	0	0	0.01	1	3642.96	2814.7
HMGB-1 (ng/mL)	92.67	7.33	0.42	0.28	−0.15	0.96	1	3536.52	2048.85
EVs (events/µL)	4.65	95.35	−10,038.83	5802.75	−21,576.23	1638.06	1	6796.11	3067.23
TF^+^ EVs (events/µL)	0	100	−1543.5	479.62	−2716.92	−813.98	1.38	8.97	13.71
CRP^+^ EVs (events/µL)	0.08	99.92	−6998.63	2129.1	−11,234.85	−2893.5	1	6375.93	3141
CRP (mg/L)	75.8	24.2	0.65	0.93	−1.14	2.52	1	3608.36	2938.3
PCT (ng/mL)	53.42	46.58	0	0.03	−0.06	0.06	1	2454.24	2441.22
IL-1β (pg/mL)	99.98	0.02	0.1	0.03	0.04	0.16	1	2596.99	2760.37
IL-6 (pg/mL)	40.95	59.05	−1.7	7.5	−16.63	13.11	1	4165.65	3158.01
IL-8 (pg/mL)	100	0	2.84	0.51	1.85	3.83	1	3460.46	2930.64
IL-10 (pg/mL)	95.95	4.05	0.15	0.09	−0.02	0.33	1	3720.19	3263.86
IFN-γ (pg/mL)	71.25	28.75	0.13	0.22	−0.31	0.56	1	2979.59	2620.35
TNF-α (pg/mL)	97.97	2.03	0.71	0.35	0.04	1.39	1	3549.91	2730.32
IP-10 (pg/mL)	0	100	−171.03	29.29	−228.73	−112.8	1	1797.06	2200.2
MCP-1 (pg/mL)	69.5	30.5	2.08	4.1	−6.18	10	1	2603.32	2691.08
G-CSF (pg/mL)	99.72	0.28	6.87	2.6	1.69	11.77	1	3776.26	3357.83

**Table 3:** Antibodies and fluorochrome conjugates used for flow cytometry.

Flow Cytometry						
Antigen	Origin	Clone	Marker for	Fluorochrome	Abbreviation	Supplier
CD41	mouse	P2	platelets	Phycoerythrin Cyanin 7	PC7	Beckman Coulter
CD235a	mouse	HIR2 (GA-R2)	red blood cells	Fluorescein Isothiocyanate	FITC	eBioscience
CD105	mouse	266	endothelial cells	Phycoerythrin	PE	Becton Dickinson
CD45	mouse	J33	leukocytes	Pacific Blue	PB	Beckman Coulter
CD36	mouse	5-271	platelet glycoprotein IV	Alexa Fluor 700	AF700	BioLegend
PF4	mouse	170138	platelet factor 4	Phycoerythrin	PE	R&D Systems
CRP	goat	-	C-reactive protein	Fluorescein Isothiocyanate	FITC	Abcam
TF	mouse	VD8	tissue factor	Fluorescein Isothiocyanate	FITC	BioMedica Diagnostics
HMGB-1	mouse	115603	high mobility group box-1 protein	Alexa Fluor 700	AF700	R&D Systems
Anx5	-	-	phosphatidylserine	Allophyco cyanin	APC	BD Biosciences

## Data Availability

Data are available on request from the authors.

## Supplementary Materials

The supporting information can be downloaded at: https://www.mdpi.com/article/10.3390/ijms242316886/s1.
