# Supplementary material for: Expression of Tissue Factor and Platelet/Leukocyte Markers on Extracellular Vesicles Reflect Platelet–Leukocyte Interaction in Severe COVID-19
Source: Int J Mol Sci. 2023 Nov 28;24(23):16886. doi: 10.3390/ijms242316886 (PMC10707108; doi:10.3390/ijms242316886)
Supplement: Supplementary file 1 [file ijms-24-16886-s001.zip › ijms-2684625-supplementary.pdf]

Supplementary Material

Expression of Tissue Factor and Platelet/Leukocyte Markers on Extracellular Vesicles Reflect Platelet–Leukocyte Interaction in Severe COVID-19

Tanja Eichhorn[1,*,†], René Weiss[1,†], Silke Huber[2], Marie Ebeyer-Masotta[1], Marwa Mostageer[1], Robert Emprechtinger[3], Ludwig Knabl, Sr.[4], Ludwig Knabl[5], Reinhard Würzner[2], Viktoria Weber[1]

[1] Center for Biomedical Technology, Department for Biomedical Research, University for Continuing Education Krems, 3500 Krems, Austria

[2] Institute of Hygiene and Medical Microbiology, Medical University of Innsbruck, 6020 Innsbruck, Austria

[3] Faculty of Health and Medicine, University for Continuing Education Krems, 3500 Krems, Austria

[4] Department of Internal Medicine, Hospital St. Vinzenz, 6511 Zams, Austria

[5] TyrolPath, 6511 Zams, Austria

[*] Corresponding author. Dr.-Karl-Dorrek-Strasse 30, 3500 Krems, Austria. Tel.: +43 2732 893 2612; e-mail: tanja.eichhorn@donau-uni.ac.at

[†] Equally contributing first authors

Supplementary Table S1. Medication regimens for individual patients.

	Medication
**Patient 1**	Concor 5 mg, Neprilisin 49/51/51 mg, Euthyrox 112 µg, Synjardy 12.5/850 mg, Trajenta 5 mg, Lasix 40 mg, Eliquis 5 mg
**Patient 2**	Amlodipin 5 mg, Aglandin Retard 0.4 mg
**Patient 3**	Pantoloc 40 mg, ThromboAss 100 mg, Thyrex 0.16 mg, Ascalan
**Patient 4**	Ramipril 2.5 mg, Ezerosu 10/20 mg, Lasilacton 20/100 mg, Bisoprolol 2.5 mg, Pantoloc 40 mg, Trittico 150 mg, Dominal forte 80 mg, Seropram 20 mg, Xarelto 20 mg
**Patient 5**	Nomexor 5 mg, Finasterid 5 mg, Tamsulosin 0.4 mg, Simvastatin 40 mg, Alendronic acid 70 mg (1x/week), Eliquis 5 mg
**Patient 6**	Acecomb mite, Concor 3.75 mg, Amlodipin 5 mg, Mucobene 600 mg, Halcion 0.25 mg, Pradaxa 110 mg
**Patient 7**	Blopress 32/12.5 mg, Concor 10 mg, Spirono 50 mg, Seropram 20 mg, Sintrom
**Patient 8**	Tamsulosin 0.4 mg, Lasilacton 20/50 mg, Magnonorm 365 mg, Quetialan 100 mg, Folsan 5 mg
**Patient 9**	Pantoloc 40 mg, Anoro Ellipta 55/22 µg
**Patient 10**	ThromboASS 100 mg, Bisocor 10 mg, Pariet 20 mg, Thyrex 100 µg
**Patient 11**	Gabapentin 300 mg, Allopurinol 100 mg, Sortis 20 mg, Eucreas 50/1000 mg
**Patient 12**	Allopurinol 300 mg, Metohexal 95 mg, Tritace 10 mg, Atorvastatin 40 mg, Lasix 30 mg, ThromboASS 100 mg, Doxazosin 4 mg

Supplementary Table S2. MIFlowCyt-EV framework.

Framework Criteria	What to report	Please complete each criterion
1.1 Preanalytical variables conforming to MISEV guidelines.	Preanalytical variables relating to EV sample including source, collection, isolation, storage, and any others relevant and available in the performed study.	All relevant pre-analytical variables are given in the Methods Section.
1.2 Experimental design according to MIFlowCyt guidelines.	EV-FC manuscripts should provide a brief description of the experimental aim, keywords, and variables for the performed FC experiment(s) using MIFlowCyt checklist criteria: 1.1, 1.2, and 1.3, respectively. Template found at www.evflowcytometry.org.	The experimental aim (to detect TF^{+} EVs carrying both platelet and leukocyte markers, mirroring enhanced platelet-leukocyte aggregate formation in severe COVID-19 patients) and the relevant variables are described in the Introduction and Methods Section of our manuscript. The relevant keywords are given.
2.1 Sample staining details	State any steps relating to the staining of samples. Along with the method used for staining, provide relevant reagent descriptions as listed in MIFlowCyt guidelines (Section 2.4 Fluorescence Reagent(s) Descriptions).	The detailed staining protocol is provided in the Methods Section of the manuscript. Fluorochrome conjugates are listed in Table 3.
2.2 Sample washing details	State any steps relating to the washing of samples.	Not required
2.3 Sample dilution details	All methods and steps relating to sample dilution.	Please refer to the Methods Section.
3.1 Buffer alone controls.	State whether a buffer-only control was analyzed at the same settings and during the same experiment as the samples of interest. If utilized it is recommended that all samples be recorded for a consistent set period of time e.g. 5 minutes, rather than stopping analysis at a set recorded event count e.g. 100,000 events. This allows comparisons of total particle counts between controls and samples.	Please refer to Supplementary Figure S4 for buffer-only controls. Buffer-only controls were used for setting up the protocol only.
3.2 Buffer with reagent controls.	State whether a buffer with reagent control was analyzed at the same settings, same concentrations, and during the same experiment as the samples of interest. If used state what the results were.	Please refer to Supplementary Figure S4 for buffer with reagent controls. Buffer with reagent controls were used for setting up the protocol only.
3.3 Unstained controls.	State whether unstained control samples were analyzed at the same settings and during the same experiment as stained samples. If used, state what the results were, preferably in standard units.	See point 3.2.
3.4 Isotype controls.	The use of isotype controls is applicable to immunofluorescence labelling only. State whether isotype controls were analyzed at the same settings and during the same experiment as stained samples. If utilized, state which antibody they are matched to, the concentration used, and what the results were (Section 4.2, 4.3, 4.4). Due to conjugation differences between manufacturers if should be stated if the isotype controls are from the same manufacturer as the matched antibodies.	Isotype controls for the flow cytometric characterization of EVs are shown in Supplementary Figure S4. They were used for establishing the protocol only.
3.5 Single-stained controls.	State whether single-stained controls were included. If used state whether the single-stained controls were recorded using the same settings, dilutions, and during the same experiment as stained samples and state what the results were, preferably in standard units (Section 4.2, 4.3, 4.4).	See point 3.2.

3.6 Procedural controls.	State whether procedural controls were included. If used, state the procedure and if the procedural controls were acquired at the same settings and during the same experiment as stained samples.	Not required
3.7 Serial dilutions.	State whether serial dilutions were performed on samples and note the dilution range and manner of testing. The fluorescence and/or scatter signal intensity would ideally be reported in standard units (see Section 4.3, 4.4) but arbitrary units can also be used. This data is best reported by plotting the recorded number events/concentration over a set period of time at different sample dilution. The median fluorescence intensity at each of the dilutions should also ideally be plotted on the same or a separate plot.	Serial dilutions were performed to determine the optimal dilution in order to avoid "swarm detection".
3.8. Detergent treated EV-samples	State whether samples were detergent treated to assess lability. If utilized, state what detergent was used, the end concentration of the detergent, and what the results were of the lysis.	The presence of intact EVs was confirmed by detergent lysis with 0.25% Triton-X 100.
4.1 Trigger Channel(s) and Threshold(s).	The trigger channel(s) and threshold(s) used for event detection. Preferably, the fluorescence calibration (Section 4.3) and/or scatter calibration (Section 4.4) should be used in order to report the trigger channel(s) and threshold(s) in standardized units.	As trigger channel Violet SS was used with a threshold of 2000 (H).
4.2 Flow Rate / Volumetric quantification.	State if the flow rate was quantified/validated and if so, report the result and how they were obtained.	The flow rate was routinely calibrated with the built-in software application "Calibrate Sample Flow Rate" of the CytoFLEX LX device.
4.3 Fluorescence Calibration.	State whether fluorescence calibration was implemented, and if so, report the materials and methods used, catalogue numbers, lot numbers, and supplied reference units for the standards. Fluorescence parameters may be reported in standardized units of MESF, ERF, or ABC beads. The type of regression used, and the resulting scatter plot of arbitrary data vs standard data for the reference particles should be supplied.	Quality control beads (CytoFLEX Daily QC Fluorospheres; Ref. B53230, Beckman Coulter) were run prior to each measurement.
4.4 Light Scatter Calibration.	State whether and how light scatter calibration was implemented. Light scatter parameters may be reported in standardized units of nm2, along with information required to reproduce the model.	See Methods Section<br><br>Calibration was performed with fluorescent silica beads (0.1 µm, 0.5 µm, 1.0 µm; excitation/emission 485/510; Kisker Biotech).
5.1 EV diameter/surface area/volume approximation.	State whether and how EV diameter, surface area, and/or volume has been calculated using FC measurements.	Not performed
5.2 EV refractive index approximation.	State whether the EV refractive index has been approximated and how this was done.	Not performed
5.3 EV epitope number approximation.	State whether EV epitope number has been approximated, and if so, how it was approximated.	Not performed
6.1 Completion of MIFlowCyt checklist.	Complete MIFlowCyt checklist criteria 1 to 4 using the MIFlowCyt guidelines. Template found at www.evflowcytometry.org.	Not completed

6.2 Calibrated channel detection range	If fluorescence or scatter calibration has been carried out, authors should state whether the upper and lower limits of a calibrated detection channel were calculated in standardized units. This can be done by converting the arbitrary unit scale to a calibrated scaled, as discussed in Section 4.3 and 4.4, and providing the highest unit on this scale and the lowest detectable unit above the unstained population. The lowest unit at which a population is deemed 'positive' can be determined a variety of ways, including reporting the 99th percentile measurement unit of the unstained population for fluorescence. The chosen method for determining at what unit an event was deemed positive should be clearly outlined.	Gates were adjusted manually related to the unstained samples or the respective isotype controls.
6.3 EV number/concentration.	State whether EV number/concentration has been reported. If calculated, it is preferable to report EV number/concentration in a standardized manner, stating the number/concentration between a set detection range.	Please refer to Results Section as well as Supplementary Figures S1.
6.4 EV brightness.	When applicable, state the method by which the brightness of EVs is reported in standardized units of scatter and/or fluorescence.	Not performed
7.1. Sharing of data to a public repository.	Provide a link to the experimental data in a public data repository.	Data will be shared at request.

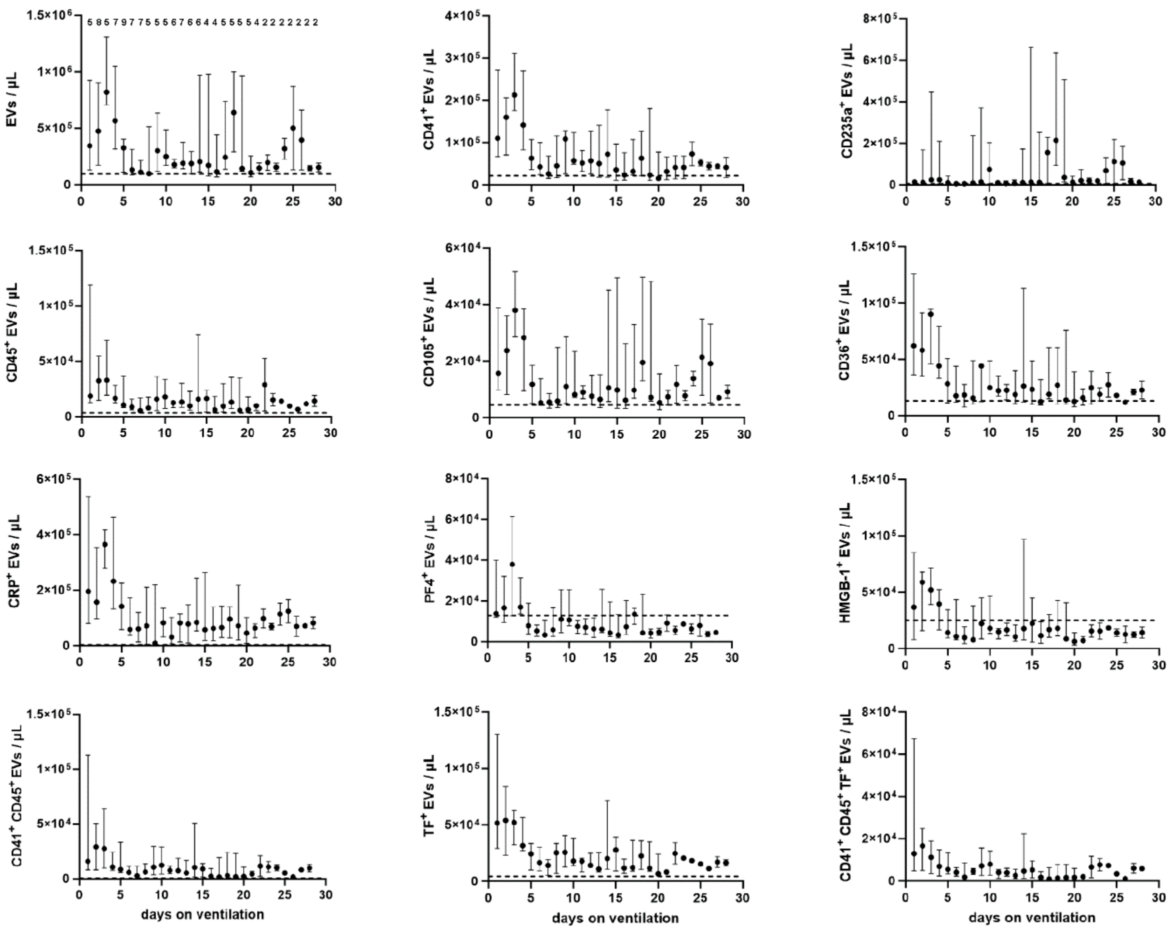

Supplementary Figure S1. Time course of extracellular vesicles (EVs) in COVID-19 patients. The dashed lines indicate reference values for healthy controls. CD235a^{+} EVs, CRP, CD41^{+}CD45^{+} EVs, and CD41^{+}CD45^{+}TF^{+} EVs were below the limit of detection in controls. The numbers in the left upper graph refer to the numbers of samples (n) for each time point. Data are given as median and interquartile range.

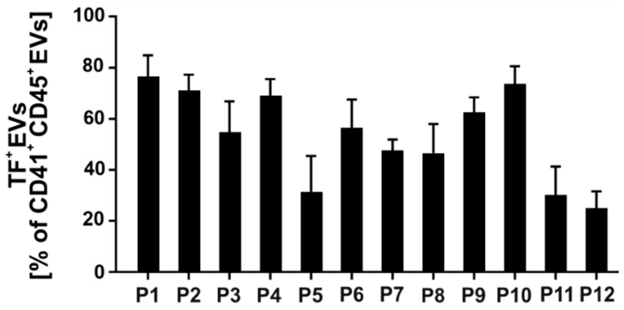

Supplementary Figure S2. Tissue factor (TF)-positive extracellular vesicle aggregates ($CD41^{+}CD45^{+}$) for individual COVID-19 patients. Data are given as median and interquartile range.

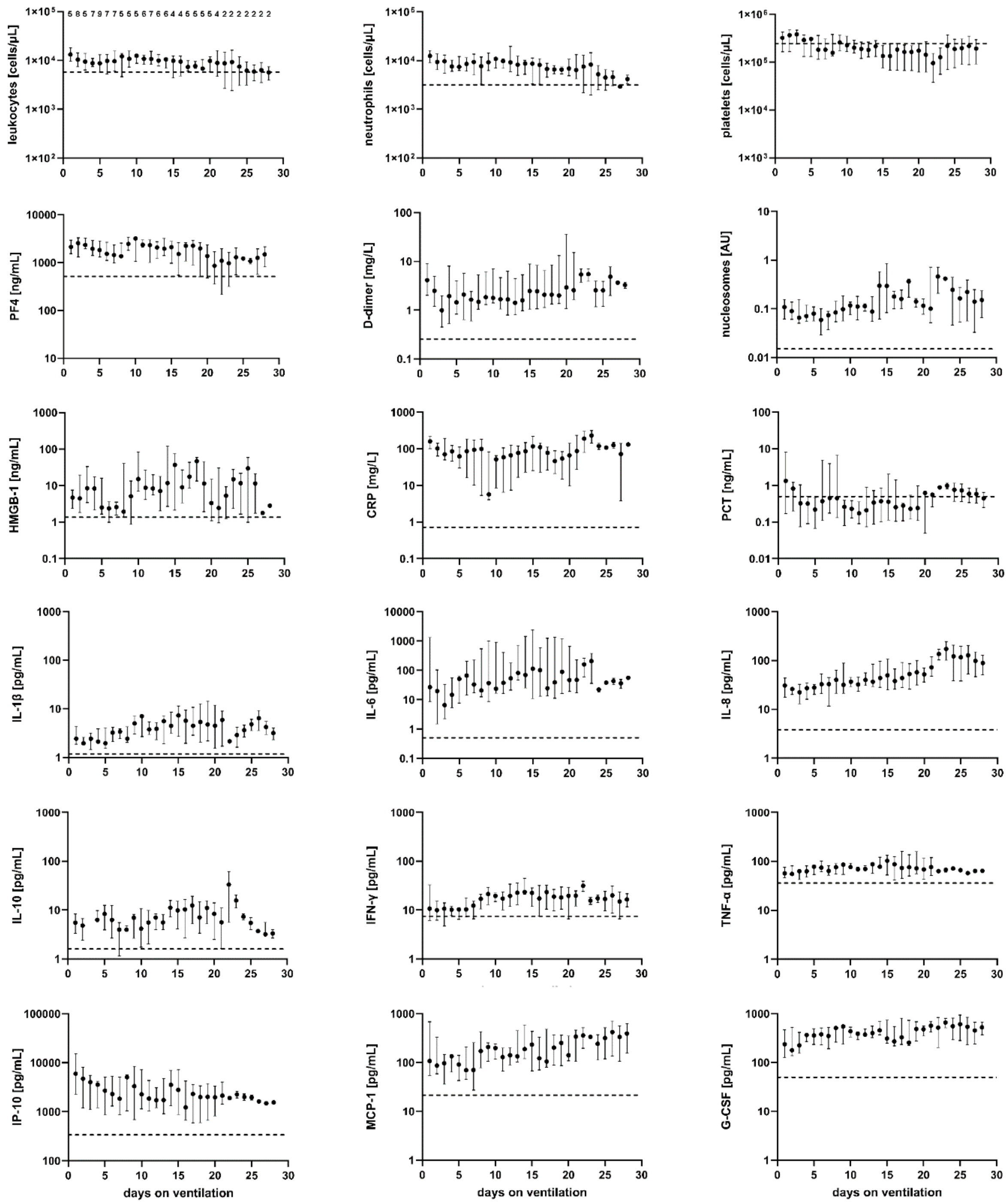

Supplementary Figure S3. Time course of inflammatory mediators in mechanically ventilated COVID-19 patients. The dotted lines indicate reference values measured in healthy controls. The numbers in the left upper graph refer to the numbers of samples (n) for each time point. Data are given as median and interquartile range.

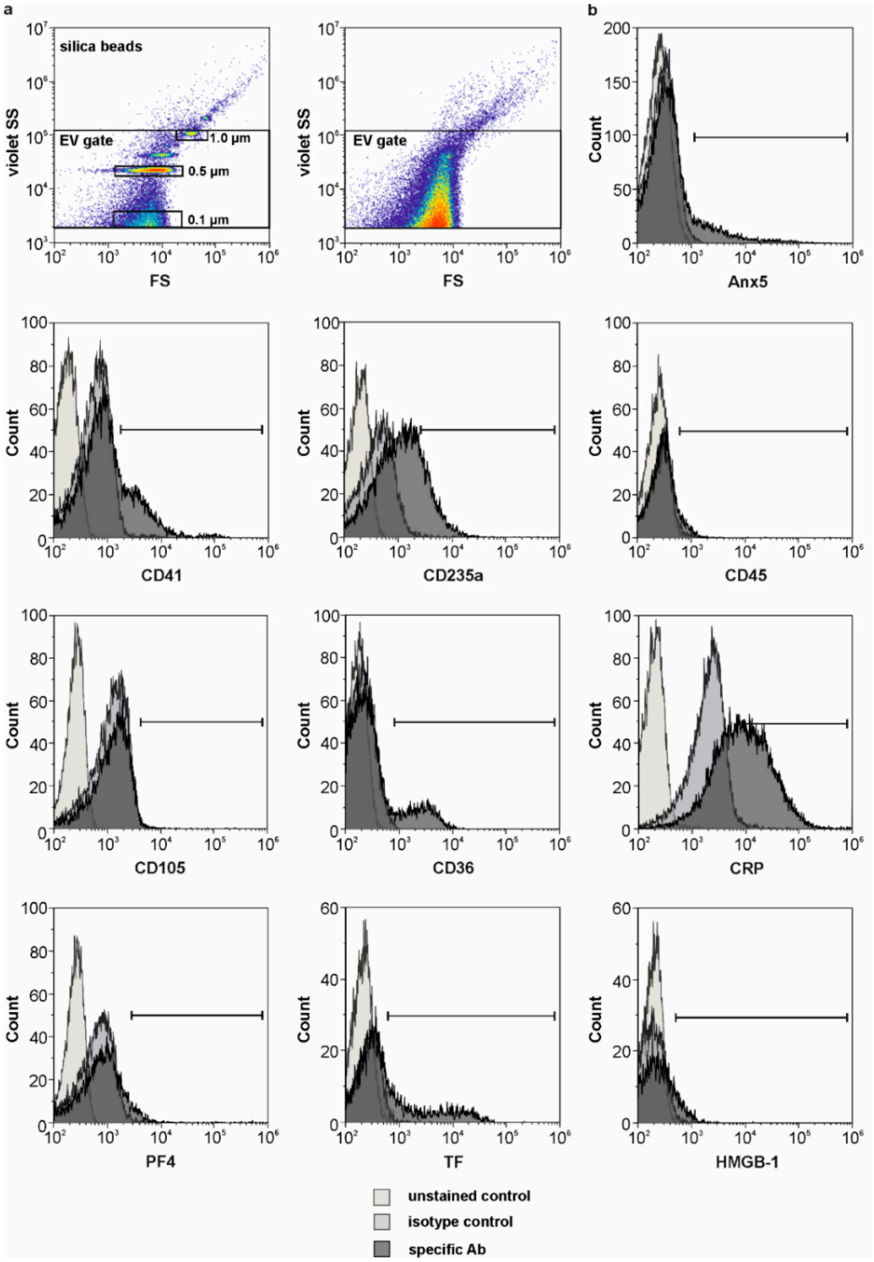

Supplementary Figure S4. Calibration and controls for the flow cytometric characterization of phosphatidylserine-exposing extracellular vesicles. (a) Flow cytometric characterization was performed on a CytoFLEX LX Flow Cytometer (Beckman Coulter) after calibration with fluorescent silica particles (0.1, 0.5, and 1.0 µm), and the EV gate was set below the 1 µm bead cloud as described in the Methods section of the main manuscript (left panel). Staining of EVs was performed as described in the Methods section and a representative forward scatter *vs*. violet side scatter (FS *vs.* violet SS) density plot (right panel) is shown. (b) The respective unstained controls, isotype controls, and single stainings are shown. Anx5 staining in PBS medium without Ca^{++} and Mg^{++} was used as negative control. Bars indicate positive expression.

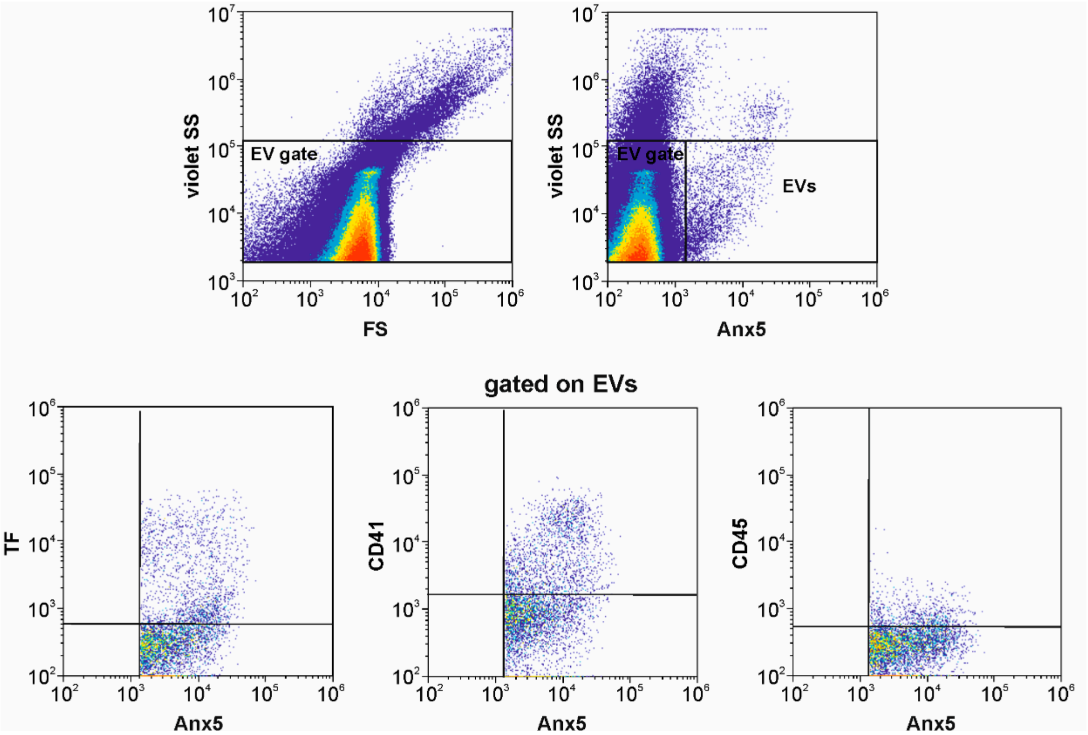

Supplementary Figure S5. Characterization of TF-positive phosphatidylserine-exposing extracellular vesicles in COVID-19 patients. To detect TF on the surface of EVs using flow cytometry, plasma samples were stained with a combination of PC7-conjugated anti-CD41 as platelet marker, PB-conjugated anti-CD45 as leukocyte marker, as well as FITC-conjugated anti-hTF to detect TF as described in the Methods section of the main manuscript. Anx5 was used as marker for EVs exposing phosphatidylserine and EVs were defined as $Anx5^+$ events in the EV gate. Representative forward scatter *vs*. violet side scatter (FS *vs.* violet SS), Anx5 *vs.* violet SS, Anx5 *vs.* TF, Anx5 *vs.* CD41, and Anx5 *vs.* CD45 density plots are shown.
